# Citrus *β*-carotene hydroxylase 2 (BCH2) participates in xanthophyll synthesis by catalyzing the hydroxylation of *β*-carotene and compensates for BCH1 in citrus carotenoid metabolism

**DOI:** 10.1093/hr/uhac290

**Published:** 2022-12-30

**Authors:** Yingzi Zhang, Jiajing Jin, Shenchao Zhu, Quan Sun, Yin Zhang, Zongzhou Xie, Junli Ye, Xiuxin Deng

**Affiliations:** Key Laboratory of Horticultural Plant Biology (Ministry of Education), Huazhong Agricultural University, Wuhan, 430070, China; Key Laboratory of Horticultural Plant Biology (Ministry of Education), Huazhong Agricultural University, Wuhan, 430070, China; Key Laboratory of Horticultural Plant Biology (Ministry of Education), Huazhong Agricultural University, Wuhan, 430070, China; Key Laboratory of Horticultural Plant Biology (Ministry of Education), Huazhong Agricultural University, Wuhan, 430070, China; Key Laboratory of Horticultural Plant Biology (Ministry of Education), Huazhong Agricultural University, Wuhan, 430070, China; Key Laboratory of Horticultural Plant Biology (Ministry of Education), Huazhong Agricultural University, Wuhan, 430070, China; Key Laboratory of Horticultural Plant Biology (Ministry of Education), Huazhong Agricultural University, Wuhan, 430070, China; Key Laboratory of Horticultural Plant Biology (Ministry of Education), Huazhong Agricultural University, Wuhan, 430070, China

## Abstract

As an essential horticultural crop, *Citrus* has carotenoid diversity, which affects its aesthetic and nutritional values. *β*,*β*-Xanthophylls are the primary carotenoids accumulated in citrus fruits, and non-heme di-iron carotene hydroxylase (BCH) enzymes are mainly responsible for *β*,*β*-xanthophyll synthesis. Previous studies have focused on the hydroxylation of *BCH1*, but the role of its paralogous gene in citrus, *BCH2*, remains largely unknown. In this study, we revealed the β-hydroxylation activity of citrus BCH2 (CsBCH2) for the first time through the functional complementation assay using *Escherichia coli*, although CsBCH2 exhibited a lower activity in hydroxylating *β*-carotene into *β*-cryptoxanthin than citrus BCH1 (CsBCH1). Our results showed that overexpression of *CsBCH2* in citrus callus increased xanthophyll proportion and plastoglobule size with feedback regulation of carotenogenic gene expression. This study revealed the distinct expression patterns and functional characteristics of two paralogous genes, *CsBCH1* and *CsBCH2*, and illustrated the backup compensatory role of *CsBCH2* for *CsBCH1* in citrus xanthophyll biosynthesis. The independent function of *CsBCH2* and its cooperative function with *CsBCH1* in* β*-cryptoxanthin biosynthesis suggested the potential of *CsBCH2* to be employed for expanding the synthetic biology toolkit in carotenoid engineering.

## Introduction

Carotenoids are the most widely distributed isoprenoid pigments, and they are synthesized by photosynthetic organisms and multiple non-photosynthetic microorganisms [1]. The prominent role of carotenoids in horticultural crops is responsible for the exterior quality of fruits and vegetables. Carotenoids also serve as vitamin A precursors and potent antioxidants with health benefits for humans [2, 3]. In nature, carotenoids are classified into two categories: carotenes and xanthophylls. In higher plants, the composition of xanthophylls such as lutein, β-cryptoxanthin, and zeaxanthin is remarkably conserved, with oxygen atoms in the structure [4, 5]. Xanthophylls are essential to plant photosynthesis. For example, the xanthophyll cycle influences the efficiency of absorbed light energy transfer to the PSII reaction center [6]. Furthermore, xanthophylls are beneficial to human health. Previous studies have shown that xanthophylls such as lutein and β-cryptoxanthin can effectively prevent eye diseases and inflammation, with high antioxidant activity [4, 7].

One of the main xanthophylls found in nature is β-cryptoxanthin, which serves as the precursor of vitamin A in humans. β-Cryptoxanthin is not commonly accumulated in horticultural crops, and it is only rich in a few fruits, such as citrus, papaya, and persimmon [8]. Citrus, as one of the essential fruits, is a plentiful source of carotenoids. The carotenoid diversity of citrus fruits is higher than that of other carotenoid-rich crops, such as tomato and carrot. Nutritional values and exterior qualities of citrus cultivars vary, with different carotenoid compositions and contents. β,β-Xanthophylls are the primary carotenoids accumulated in citrus fruits and flowers, and they are responsible for the color. According to carotenoid diversity, citrus cultivars are divided into three categories, namely, β-cryptoxanthin-rich cultivars, violaxanthin-rich cultivars, and low-content carotenoid cultivars. The fruit of mandarins exhibits a higher level of β-cryptoxanthin than that of oranges, accounting for >90% of the total carotenoids, and oranges primarily accumulate violaxanthin in the pulp [9–11]. The massive accumulation of β-cryptoxanthin endows citrus fruits with high nutritional value, and citrus fruits act as the major human dietary β-cryptoxanthin source. Therefore, revealing the biosynthesis mechanism of β-cryptoxanthin in citrus fruits is helpful for their quality improvement.

Key genes involved in carotenoid synthesis, such as *PSY1* and *LCYB*, have been isolated, and their functions in citrus fruit have been thoroughly investigated and applied to the engineering of carotenoid synthesis metabolism [9, 12–15]. Carotene hydroxylation is an important part of carotenoid synthesis. Hydroxylation of the carotenoid rings is catalyzed by ring-specific hydroxylase, and it can convert carotenes into xanthophylls such as lutein and zeaxanthin [16]. Two types of carotene hydroxylases have been reported in plants, including non-heme di-iron carotene hydroxylase (also known as BCH, HYD, or HYb) and heme-containing cytochrome P450 (CYP) carotene hydroxylase [7]. BCH can efficiently catalyze the hydroxylation of the *β*-rings of β-carotene. The first β-carotene hydroxylase (named CrtZ) was detected in the phytopathogenic bacterium *Erwinia uredovora* [17]. Two *BCH* genes have been characterized in *Arabidopsis thaliana*, and they display 30–37% sequence identity with *CrtZ*. The double-null mutation of *BCH1* and *BCH2* has been reported to result in a significant decrease in β,β-xanthophylls [5, 18, 19]. Overexpression of *BCH1* increases xanthophyll level but decreases β-carotene level in *Arabidopsis*, tomato, carrot, and kiwifruit [20–23]. Silencing *CHY1* and *CHY2* in potato tubers can increase β-carotene content and total carotenoid content, with a significantly better effect achieved by silencing *CHY1* than silencing *CHY2* [24]. A similar observation has been reported in citrus, that BCH1 can hydroxylate carotenes into β-cryptoxanthin and zeaxanthin in *Escherichia coli* and that silencing BCH1 can increase carotene content, but total carotenoid content is decreased, with β-xanthophylls remaining at the highest proportion in carotenoids [25]. The existing studies of β-carotene hydroxylation have primarily focused on the function of BCH1, and revealed that BCH1 can catalyze β-carotene hydroxylation [7]. However, the functions of *BCH2* (paralogous gene of *BCH1*) in citrus carotenoid metabolism remain largely unknown.

Phylogenetic analyses have indicated that *BCH* gene duplication events occurred after the monocot–dicot split in higher plants [5]. Gene duplication and subsequent functional divergence induced by mutations in protein-coding regions and/or gene expression pattern change are increasingly recognized as crucial evolutionary mechanisms [5]. Previous studies have demonstrated that two BCH members in some higher plants present similar functions, although they are specifically expressed in different tissues [5, 20, 26]. Examining the functional characteristics of citrus *BCH2* (named *CsBCH2*) is vital for revealing xanthophyll accumulation in citrus fruit and the evolutionary mechanism of paralogous genes. To determine whether *CsBCH2* plays the same role as citrus *BCH1* (named *CsBCH1*) in carotenoid synthesis, we analyzed the expression patterns and functional characteristics of *CsBCH2 in vivo* and *in vitro*. The functional complementation assay showed that CsBCH2 could hydroxylate β-carotene into β-cryptoxanthin in *E. coli*. Results of analysis of the expression pattern and functional characteristics of *CsBCH2* revealed that it played an essential role in citrus xanthophyll biosynthesis, meanwhile providing backup compensation for *CsBCH1*.

## Results

### Isolation and sequence analysis of *CsBCH2*

**Figure 1:**
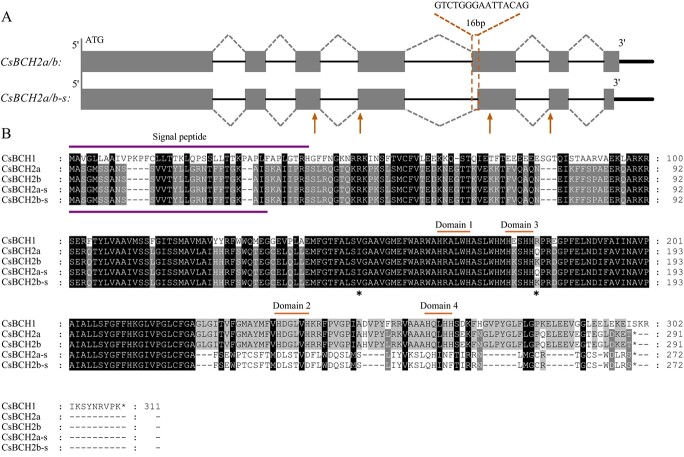
Gene structures of *CsBCH2* alleles and protein sequence alignments of CsBCH1 and CsBCH2. (A) Schematic of *CsBCH2* transcript structures. Gray boxes represent exons, black lines indicate introns, bold black lines indicate untranslated regions, orange arrow indicates the position of conserved domain, and the orange dashed box represents the alternative splicing region. (B) Amino acid sequence alignments. Signal peptides and the conserved domains are marked by purple lines and orange lines. Asterisks indicate non-synonymous sites.

With the continuous release of citrus genomic and transcriptomic data, *BCH2* (the paralogous gene of *BCH1*), has gradually attracted our attention. Given the genetic diversity of citrus species, we isolated *BCH*2 from the cDNA of three common citrus species fruits, including mandarin (*Citrus reticulata* Blanco), sweet orange (*C. sinensis* Osbeck), and pummelo [*C. maxima* (Burm.) Merr.] and named it *CsBCH2*, to explore its catalytic activity. Two *CsBCH2* alleles (*CsBCH2a* and *CsBCH2b*) were obtained from chromosome 5. Both *CsBCH2a* (from mandarin and sweet orange) and *CsBCH2b* (from pummelo and sweet orange) contained an 876-bp open reading frame (ORF) and encoded one putative 32.4-kDa protein consisting of 291 amino acids with two non-synonymous sites. We also obtained two different transcripts corresponding to the two alleles (*CsBCH2a-s* and *CsBCH2b-s*). These two transcripts encoded a 272-amino acid protein and carried a 16-bp alternative splicing (Fig. 1A). The putative protein encoded by *CsBCH2a*/*b* contained highly conserved histidine domains, including two ‘HXXXXH’ domains (Domain 1 ‘HRALWH’ and Domain 2 ‘HDGLVH’) and two ‘HXXHH’ domains (Domain 3 ‘HKSHH’ and Domain 4 ‘HQLHH) (Fig. 1B), which was consistent with the domains of *BCH* genes in carrot, pepper, and kiwifruit [23, 27, 28]. Alternative splicing of *CsBCH2a-s* and *CsBCH2b-s* occurred at 646 bp downstream of the translational start codon of the cDNA sequence, thereby causing the frameshifts of the ‘HDGLVH’ and ‘HQLHH’ conserved domains, which might affect the catalytic activity of the enzyme.

To characterize *CsBCH2*, the coding sequence of *CsBCH1* on chromosome 9 was obtained for subsequent comparative analysis. *CsBCH1* was 936 bp in length, and it encoded a 34.7-kDa protein with 311 amino acids (Fig. 1B), with 69% amino acid sequence identity with CsBCH2a/b. CsBCH1 contained the same two conserved histidine domains, ‘HDGLVH’ (Domain 2) and ‘HQLHH’ (Domain 4), as CsBCH2. Unlike CsBCH2, the other two conserved domains, ‘HKALWH’ and ‘HESHH’ of CsBCH1 contained two non-synonymous sites. The above results showed similar sequences and conserved domains between CsBCH1 and CsBCH2, implying that the two BCHs might have similar functions in citrus.

### Expression patterns of *CsBCH* genes in citrus

The transcriptome data published on the Citrus Pan-genome to Breeding Database (http://citrus.hzau.edu.cn/) showed that *CsBCH1* and *CsBCH2* transcript levels differed among different tissues and species (Supplementary Data Fig. S1). Further, we identified the spatial and temporal expression patterns of *CsBCH1* and *CsBCH2* using quantitative real-time PCR (qRT–PCR) in two carotenoid-rich citrus species, including β-cryptoxanthin-rich cultivar Ponkan mandarin and violaxanthin-rich cultivar Hamlin sweet orange [11].

**Figure 2:**
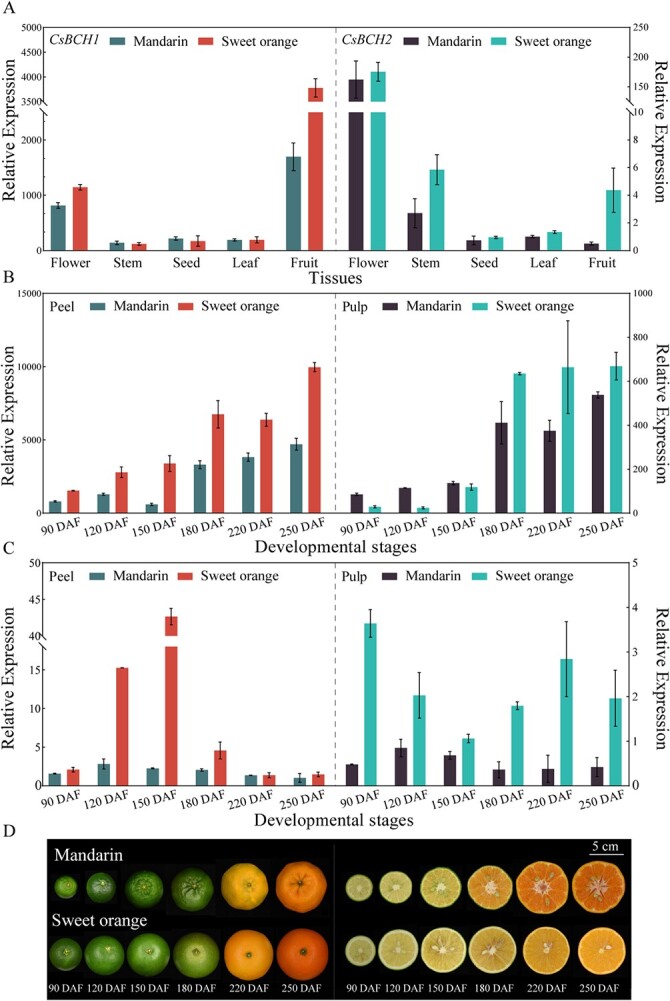
Expression patterns of *CsBCH* genes in citrus fruit. (A) *CsBCH* gene expression patterns were compared between mandarin (Ponkan) and sweet orange (Hamlin) in different tissues by qRT–PCR. (B, C) Relative expression levels of *CsBCH1* (B) and *CsBCH2* (C) at six development stages of citrus peel and pulp. (D) Phenotype of fruit at six development stages. DAF, days after flowering. Data are mean ± standard error of triplicate samples. Scale bar = 5 cm.

The qRT–PCR results showed that *CsBCH1* was highly expressed in flowers and fruits, especially in fruits, and *CsBCH2* was mainly expressed in flowers (Fig. 2A). Both *CsBCH1* and *CsBCH2* showed tissue-specific expression in xanthophyll-accumulating tissue, and the expression levels of the two genes were higher in sweet orange than in mandarin. Given that citrus fruit is the predominant tissue for carotenoid accumulation, we performed qRT–PCR to compare *CsBCH* gene expression levels at six stages of fruit development (Fig. 2B and C). The results showed that the two *CsBCH* genes were expressed differently throughout the developmental stages of the citrus fruit. As the fruit ripened, the relative expression level of *CsBCH1* increased gradually and was higher in citrus peel, which was consistent with the β-cryptoxanthin accumulation trend in mandarin [7]. In the peel, the relative expression level of *CsBCH2* gene continued to increase in the early stages and then decreased after the color-turning stage in sweet orange. By contrast, in the pulp, it decreased first and then increased in sweet orange with violaxanthin accumulation, but the overall expression level of *CsBCH2* was lower than that of *CsBCH1*. Our results suggested that *CsBCH1* and *CsBCH2* exhibited distinct expression tissue preferences and patterns as the fruit ripened. The spatial and temporal expression patterns associated with the accumulation of xanthophylls in fruits indicated the distinct roles of these two genes in citrus.

### Functional complementation assay of CsBCH2 in *E. coli*

**Figure 3:**
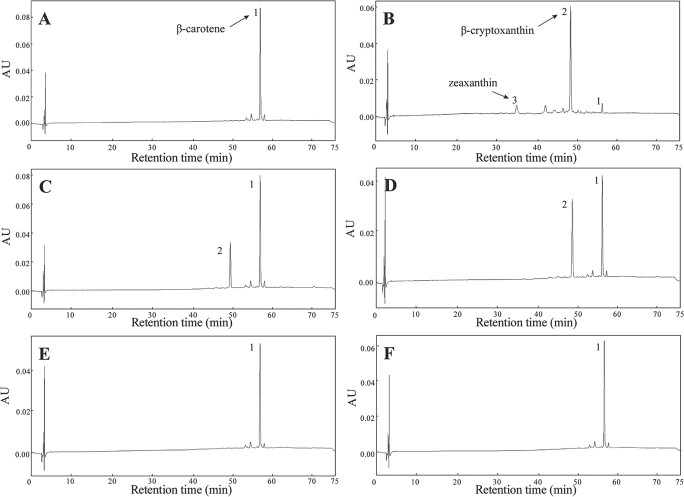
HPLC analysis of carotenoids in *E. coli* expressing *CsBCH1* or *CsBCH2*. (A–F) pHIS8 (A), pHIS8-CsBCH1 (B), pHIS8-CsBCH2a (C), pHIS8-CsBCH2b (D), pHIS8-CsBCH2a-s (E), or pHIS8-CsBCH2b-s (F) was co-transformed into *E. coli* BL21(DE3) cells with pACCAR16∆crtX. Reverse-phase HPLC was used to separate and determine carotenoids with spectra extracted at 450 nm. AU, absorbance unit; peak 1, β-carotene; peak 2, β-cryptoxanthin; peak 3, zeaxanthin.

Since the complex and dynamic metabolic network obscures plant enzyme activities, it was not easy to directly investigate substrate specificity. Functional complementation assay of enzymes *in vitro* is an effective method for exploring enzyme characteristics with *E. coli* or yeast used as a heterologous host. To characterize the enzyme more directly, we explored the activity and substrate specificity of CsBCH2 through a functional complementation assay in *E. coli*, in which four transcripts of *CsBCH2* (CsBCH2a, CsBCH2b, CsBCH2a-s, and CsBCH2b-s) were expressed in β-carotene-accumulating *E. coli*, and carotenoid products were extracted from bacteria for subsequent high-performance liquid chromatography (HPLC). The results showed that CsBCH2a- and CsBCH2b-expressing cells accumulated the substrate β-carotene and the mono-hydroxylated product *β*-cryptoxanthin, but the di-hydroxylated product zeaxanthin was undetectable. By contrast, neither *β*-cryptoxanthin nor zeaxanthin accumulation was detected in CsBCH2a-s- and CsBCH2b-s-expressing cells, suggesting that the alternative splicing-induced frameshift deprived CsBCH2 of its hydroxylation activity (Fig. 3C–F).

To better understand the characteristics of CsBCH2, we also expressed CsBCH1 in *E. coli* for comparative analysis. Consistent with the functions of BCH1 reported in other studies [7, 16], our results showed that CsBCH1 effectively hydroxylated the two* β*-rings of β-carotene, resulting in the formation of the mono-hydroxylated intermediate β-cryptoxanthin and the di-hydroxylated product zeaxanthin with a higher level of β-cryptoxanthin than zeaxanthin (Fig. 3B). Only a trace amount of β-carotene substrate was detected in the cells. All the above results showed that both CsBCH1 and CsBCH2 could hydroxylate β-carotene, and CsBCH1 exhibited a higher hydroxylation activity.

### Overexpression of *CsBCH2* in citrus callus Rm

**Figure 4:**
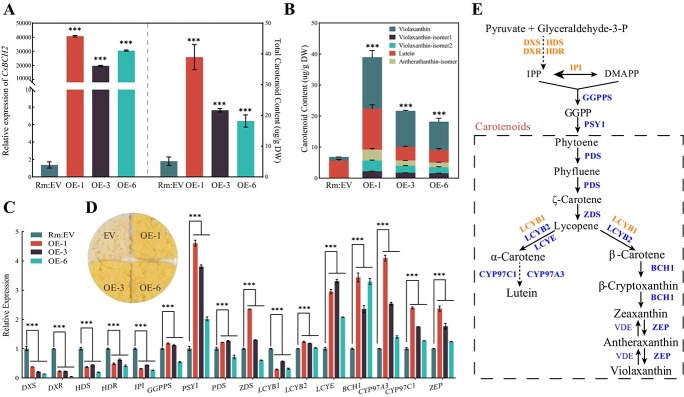
Carotenoid accumulation and carotenogenic gene expression levels in *CsBCH2*-overexpressing transgenic lines of citrus callus Rm. (A) Relative expression levels of *CsBCH2* (left) and total content of carotenoid (right) in transgenic calli Rm. (B) Composition and content of carotenoid in transgenic calli Rm. (C) Expression patterns of carotenogenic genes in transgenic lines. (D) Phenotypes of transgenic line callus Rm. (E) Schematic of general pathways of carotenoid biosynthesis in citrus. Upregulated genes in blue; downregulated genes in orange. Data are expressed as mean ± standard error for triplicate samples. Asterisks above the bars indicate statistically significant differences compared with EV (Student’s *t*-test): ^***^P < .001.

Enzyme activity and regulation in plants may not be accurately reflected in *E. coli*, which is a limitation of *in vitro* experiments. Since citrus is a perennial woody tree with a long juvenile period, it will take a long time to obtain the fruit of transgenic lines. Considering this, we explored the function of *CsBCH2* in citrus callus, a valuable *in vivo* system that has been used successfully for evaluating the function of carotenogenic genes [9, 29–31]. The full-length transcript of *CsBCH2* from mandarin was overexpressed in citrus callus Rm (white) to investigate the role of *CsBCH2* in carotenoid metabolism. The results showed that, compared with empty vector (EV) callus, the *CsBCH2*-overexpressing transgenic lines all turned light yellow. Subsequently, the carotenoid composition and content in three selected transgenic lines were determined using HPLC (Fig. 4D). The results showed that the total carotenoid content was increased in transgenic lines, and xanthophylls such as violaxanthin and violaxanthin isomers were significantly increased, which explained the callus color change (Fig. 4B, Supplementary Data Table S3).

Since the expression level of endogenous carotenogenic genes is always influenced by manipulating the carotenoid level [24], we analyzed the relative expression levels of major carotenogenic genes to reveal the mechanism of carotenoid variation in transgenic lines (Fig. 4C). The results demonstrated that the expression levels of carotenogenic genes upstream of the geranylgeranyl diphosphate (GGPP) synthesis step were inhibited (Fig. 4E). All the carotenogenic genes downstream of the carotenoid synthesis pathway (except *LCYB1*) exhibited significant upregulation; especially, the rate-limiting enzyme gene *PSY1* was upregulated >4-fold. Overall, the overexpression of *CsBCH2* increased the content of xanthophylls and jointly regulated carotenoid synthesis with other carotenogenic genes in citrus callus.

### Overexpression of *CsBCH2* in β-carotene-accumulating callus Rm33

**Figure 5:**
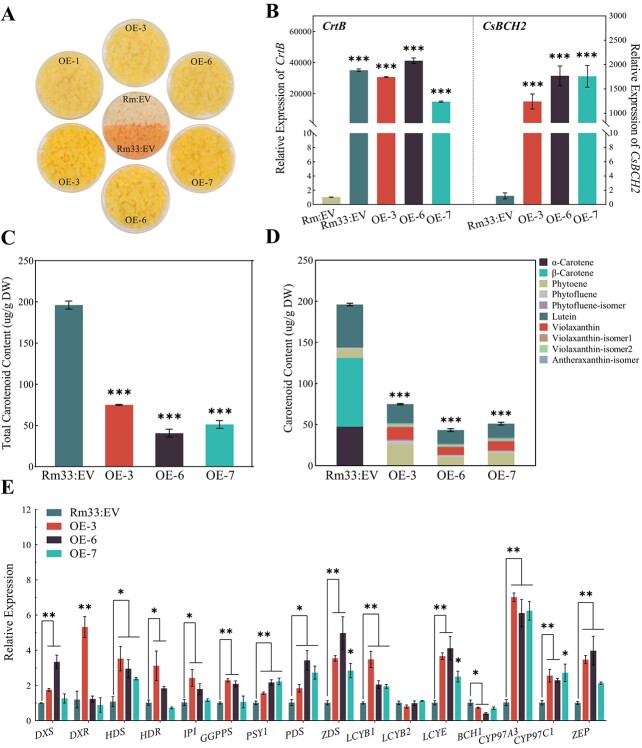
Carotenoid accumulation and carotenogenic gene expression levels in *CsBCH2*-overexpressing transgenic lines of callus Rm33. (A) Phenotypes of transgenic line callus Rm (upper part) and Rm33 (lower part). (B) Relative expression levels of *CrtB* (left) and *CsBCH2* (right) in transgenic lines. (C) Total carotenoid content in transgenic lines. (D) Composition and content of carotenoids in transgenic lines. (E) Expression levels of carotenogenic genes in transgenic lines. Data are expressed as mean ± standard error for triplicate samples. Asterisks above the bars indicate statistically significant differences compared with EV (Student’s *t*-test): ^*^P < .05; ^**^P < .01; ^***^P < .001.

Considering that β-carotene is the hydroxylated substrate of BCH, we overexpressed *CsBCH2* in the β-carotene-accumulating callus Rm33 [engineering cell model (ECM), which had already overexpressed a bacterial phytoene synthase gene (*CrtB*) in callus Rm] [29]. We selected three transgenic lines (OE-3, OE-6, and OE-7) for carotenoid content and carotenogenic gene expression analysis with the premise of not disrupting overexpression of *CrtB* in callus Rm33 (Fig. 5B). Control lines (callus Rm33 with empty vector) exhibited a high level of β-carotene (42.6%), lutein (26.6%), and α-carotene (24.2%) but only a low level of phytoene (7.4%) and phytofluene (1.5%) (Supplementary Data Table S4). The accumulation of carotenes was not detected in transgenic lines, but the percentages of oxygenated carotenoids in the three transgenic lines were increased to 57.6–69.1%. Of oxygenated carotenoids, the violaxanthin content of the β-branch in three transgenic lines was increased to 26.8–31.5%, and the lutein content of the α-branch was significantly decreased to 16.78–23.13 μg/g dry weight (DW), but as a percentage of total carotenoid content it was increased to 30.8–38.4%. Furthermore, total carotenoid content in transgenic lines (43.71–75.10 μg/g DW) was significantly lower than that in the control lines (196.09 ± 4.92 μg/g DW).

In addition to the changes in composition and content of carotenoids, our results also showed that the expression levels of all the main carotenogenic genes in transgenic lines were upregulated to varying degrees (Fig. 5E), which was consistent with the trend of the Rm overexpressing *CsBCH2* (Fig. 4C). The upregulation of carotenogenic genes upstream of the carotenoid synthesis pathway led to increases in phytoene and phytofluene contents. Notably, *LCYE*, a cyclase gene involved in α-branch synthesis, was 2- to 4-fold upregulated. *CYP97A3* was 6- to 7-fold upregulated, and *CYP97C1* was upregulated ~2-fold. By contrast, *LCYB2*, involved in β-branch synthesis, was not upregulated, and *BCH1* expression was inhibited significantly in two transgenic lines (OE-3 and OE-6). Compared with citrus callus Rm, callus Rm33 accumulated more β-branch carotenoids than α-branch carotenoids, which might be attributed to the overexpression of *CrtB* (*PSY1*). The increased proportion of lutein in total carotenoids was attributed to the upregulation of genes in α-branch synthesis, and the downregulation of *BCH1* expression level might be the regulation mechanism to balance carotenoid synthesis between the α-branch and the β-branch. The above results implied that the overexpression of *CsBCH2* in callus Rm33 could simultaneously affect the carotenoid composition and the expression level of other endogenous carotenogenic genes.

### Introduction of *CsBCH2* into citrus callus affects the plastoglobules of plastids

**Figure 6:**
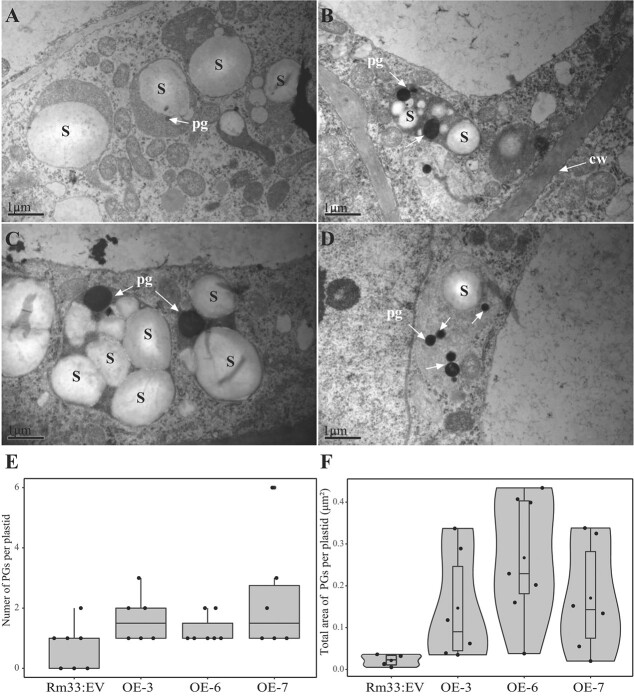
TEM of plastid ultrastructure from transgenic line callus Rm33. (A) Plastid ultrastructure of transgenic line callus Rm33 carrying the EV. (B–D) Plastid ultrastructure of transgenic callus Rm33 expressing *CsBCH2* (OE-3, OE-6, OE-7). (E) Number of PGs per plastid in transgenic lines. (F) Total area of PGs per plastid. S, starch granule; pg, plastoglobule; cw, cell wall. Scale bar = 1 μm.

Plastoglobules (PGs) are plastid lipoprotein particles found in chromoplasts, which are unique organelles responsible for synthesizing and storing large amounts of carotenoids [32, 33]. Since carotenoid variation can affect plastid morphology and plastoglobule size, we investigated plastid ultrastructure in Rm33 transgenic lines using transmission electron microscopy (TEM) to explore the effects of *CsBCH2* overexpression at the cellular level. The results showed that plastids in callus Rm33 were primarily present in the form of amyloplasts accumulating starch granules and a small number of PGs, which was consistent with the previously reported ultrastructural features of ECMs [29]. With the overexpression of *CsBCH2*, we observed that the morphology of plastids converted from globular to oval or fusiform. In addition, the *CsBCH2*-overexpressing transgenic lines exhibited amyloplast-like structures containing starch granules and more visible PGs than callus Rm33 (Fig. 6). Statistical analysis showed that although the number of PGs per plastid did not increase significantly in some cells, the total area of PGs per plastid increased among transgenic lines, which affects the capability of carotenoid storage (Fig. 6E and F). We suggest that the morphology conversion of plastids and the increase in PG size were associated with the change in carotenoid composition and the increased accumulation of xanthophylls in transgenic callus. Taking these results together, the cellular ultrastructural changes in transgenic lines indicated that the overexpression of *CsBCH2* in citrus callus could regulate the storage ability of carotenoids by affecting the carotenoid sequestration substructures.

## Discussion

Carotenoids are important secondary metabolites that are beneficial to human health and they act as colorants in most horticultural plants. The oxygenated derivative xanthophylls are the most abundant carotenoids in the light-harvesting complexes, and they serve as substrates for volatile products and precursors of hormone synthesis in plants [5]. As an important horticultural crop, citrus has a large variety of carotenoids, especially abundant β,β-xanthophylls synthesized by BCH [7]. In this study we systematically examined the hydroxylation activity of citrus CsBCH2 and elucidated the vital role of CsBCH2 in the biosynthesis of citrus xanthophyll by comparing CsBCH2 with the well-studied CsBCH1.

### CsBCH2 participates in xanthophyll synthesis by hydroxylating β-carotene

Extensive studies have shown that BCH1 can hydroxylate β-carotene [19, 26]. The β,β-xanthophylls (mainly 9-*Z*-violaxanthin) are the major component in *β-CHX* (*BCH1*)-silenced citrus [25], implying that other enzymes are also involved in β-branch hydroxylation to supply substrate for violaxanthin synthesis. *BCH2* has been reported to be differentially expressed during fruit development, thus affecting the accumulation of carotenoids [34]. Revealing the role of BCH2 is helpful for understanding xanthophyll biosynthesis in citrus.

Heterologous expression is an efficient approach to exploring enzyme characteristics *in vitro*. Expressing tomato CrtR-b1 (also named BCH1) and CrtR-b2 (also named BCH2) in β-carotene-accumulating *E. coli* confirmed that both BCH1 and BCH2 can catalyze β-carotene into β-cryptoxanthin and zeaxanthin [26]. The enzymatic activity analysis results of kiwifruit *AcBCH* genes in β-carotene-accumulating yeast showed that AcBCH1 could convert β-carotene into β-cryptoxanthin and zeaxanthin, whereas AcBCH2 could not [23]. Our *E. coli* complementary functional assay results showed that citrus CsBCH1 hydroxylated β-carotene into β-cryptoxanthin and a small amount of zeaxanthin (Fig. 3B). However, CsBCH2a and CsBCH2b hydroxylated β-carotene to generate only β-cryptoxanthin in *E. coli*, without zeaxanthin. Since no hydroxylation products were detected, we speculated that the transcripts (CsBCH2a-s and CsBCH2b-s) with frameshifts in two conserved histidine domains (Domain 2 and Domain 4) might have lost their hydroxylation activity and the highly conserved histidine domains of CsBCH2 were necessary for the hydroxylation activity (Figs 1B and 3). Similar functions of the two *CsBCH* genes did not mean that *CsBCH2* was completely redundant, since they exhibited different expression patterns in citrus. The spatial and temporal expression patterns showed that the high level of β-cryptoxanthin accumulation in citrus fruits was associated with the expression of *CsBCH1* and *CsBCH2*. The higher expression of *CsBCH1* than *CsBCH2* in fruits might be attributed to the stronger β-cryptoxanthin synthesis capability of *CsBCH1* than *CsBCH2* (Fig. 2). Compared with previous functional analyses of *BCH* genes in other horticultural crops, such as tomato and kiwifruit [23, 26], our results revealed that the specific accumulation of β-cryptoxanthin in citrus fruit might be attributed to the joint action of two *CsBCH* genes and their strong capability for β-cryptoxanthin synthesis. In addition, the higher concentration of β-cryptoxanthin in mandarin than in sweet orange might be related to the regulation of other factors, such as the participation of unreported enzymes. The differential accumulation of β-cryptoxanthin in different citrus species remains to be further investigated in future studies.

Studies of *Arabidopsis* have indicated that four enzymes (BCH1, BCH2, CYP97C1, and CYP97A3) are involved in the hydroxylation of carotenes. In spite of the fact that CYP97A can only convert β-carotene into β-cryptoxanthin in *E. coli*, only dihydroxy β-carotene derivatives (violaxanthin and neoxanthin) rather than β-cryptoxanthin are detected in the *Arabidopsis* triple mutant *bch1 bch2 cyp97c1* [5]. However, our experimental results indicated that CsBCH2 only converted β-carotene into β-cryptoxanthin in *E. coli*, which did not mean that CsBCH2 could not produce the downstream product zeaxanthin with the assistance of other factors *in vivo*. Overexpression of *CsBCH2* resulted in a higher level of accumulation of β,β-xanthophylls in transgenic callus Rm and Rm33 than in EV. The change in gene expression may be the major factor affecting carotenoid biosynthesis, and thus manipulating endogenous carotenogenic gene expression can change carotenoid content [26]. Our findings in citrus callus showed that the overexpression of *CsBCH2* could cause feedback on other carotenogenic genes and regulate the expression level of these genes to varying degrees, which jointly affects the accumulation of carotenoids in citrus callus. The upregulation of *PSY1* led to the increased total carotenoid content in transgenic callus Rm, whereas the total carotenoid content in Rm33 transgenic lines was decreased by 28% compared with that in EV, which might be attributed to the conversion of carotenes into xanthophylls and downstream hormone synthesis. These results were consistent with the previous reports that violaxanthin, rather than zeaxanthin, was accumulated with *CrtR-b2* overexpression in tomato [20], and that the contents of β-carotene and total carotenoid were decreased in leaves of *AcBCH*-overexpressing transgenic lines of kiwifruit [23]. Ultrastructure observations by TEM showed the coexistence of amyloplasts and chromoplasts in *CsBCH2*-overexpressing transgenic callus with an increase in xanthophylls, supported by the reported findings that xanthophylls could be deposited in amyloplasts, which could convert into chromoplasts in non-photosynthetic tissues [29]. The change in carotenoid composition and content in transgenic callus could affect plastid morphology and the abundance of PGs, indicating that overexpression of *CsBCH2* simultaneously influenced carotenoid storage ability at the cellular level. Although the citrus callus validation platform could not reflect the real regulation in natural fruit tissues, it simulates the composition of carotenoids in fruits and provides an abundant β-carotene substrate for β-carotene hydroxylation validation. Our study on the functional exploration of *CsBCH2 in vivo* and *in vitro* provided evidence for the pleiotropic effects of *CsBCH2* on citrus carotenoid biosynthesis.

### Hydroxylation of CsBCH2 expands the synthetic biology toolkit for carotenoid metabolic engineering

The functional complementation assay based on metabolic synthetases from various sources has turned out to be a feasible approach to diversifying metabolic biosynthesis as in nature [35–37]. With *E. coli* used as a heterologous host, the functional complementation assay of carotenogenic genes can characterize the enzymes encoded by carotenogenic genes and synthesize various carotenoids as enzyme substrates and standards [37]. Compared with that of CsBCH1, hydroxylation of CsBCH2 only converted β-carotene into β-cryptoxanthin, based on which we could construct β-cryptoxanthin-accumulating *E. coli* by expressing CsBCH2 and analyze the functions of β-cryptoxanthin-related enzymes. Therefore, this study provides the resources for functional complementation assay-based carotenoid metabolism research, especially research on β-cryptoxanthin.

Despite being time-consuming, the callus transgenic system provides an effective platform for research on carotenoid metabolism-related genes in perennial woody trees. Callus-generating carotenoid substrates such as ECMs can be employed for carotenoid studies [29]. The citrus callus used in our study provided a useful *in vivo* system for carotenoid research, and indicated that the overexpression of *CsBCH2* in citrus callus could not only regulate the expression level of other carotenogenic genes to change carotenoid biosynthesis, but also influence the number and size of plastoglobules at the cellular level. Our findings revealed the vital role of *CsBCH2* in citrus carotenoid metabolism, which could be utilized to expand the synthetic biology toolbox for carotenoid metabolism.

### Roles of two paralogous *CsBCH* genes in β-carotene hydroxylation and carotenoid metabolism

Paralogous homologs generated from gene duplication events during genome evolution facilitate genetic redundancy and phenotypic robustness [38]. Most duplicated genes are retained during evolution through multiple mechanisms such as neofunctionalization, subfunctionalization, dosage amplification, and backup compensation [39–42]. After the monocot–dicot split, *BCH* gene duplication events occurred in higher plants, such as the horticultural crops kiwifruit [23], potato [24], tomato [26], and pepper [28], containing two *BCH* paralogous genes with different tissue-specific expression patterns [5, 43]. The *AcBCH* genes (*AcBCH1* and *AcBCH2*) exhibit a constitutive expression pattern and are highly expressed in kiwifruit leaves and fruits [23]. *BCH1* (also named *CHY1*) is expressed preferentially in potato leaves, whereas *BCH2* (*CHY2*) is expressed preferentially in potato flowers [24]. In tomato, *CrtR-b1* is expressed in leaves and sepals and has low expression in petals, while *CrtR-b2* is highly expressed in the petals and anthers of flowers, where large amounts of yellow xanthophylls are accumulated [26]. Our results revealed that the expression pattern of *CsBCH1* was consistent with the high xanthophyll accumulation level during the fruit ripening process in citrus. In contrast, *CsBCH2* displayed a different expression pattern from *CsBCH1* in fruits during the color-change stage, and *CsBCH2* was preferentially expressed in flowers (Fig. 2).

Since xanthophylls play crucial roles in photosynthesis and act as precursors of abscisic acid, maintaining xanthophyll synthesis is vital to the plants themselves [6]. When *β-CHX* (*BCH1*) was silenced, β,β-xanthophylls (mainly 9-*Z*-violaxanthin) remained as the primary carotenoid in citrus [25]. Comparison of β-ring hydroxylation between the *b1 b2* double mutant and the *b1* mutant has demonstrated that BCH2 can largely compensate for the absence of BCH1 activity in mutant *Arabidopsis* [19]. Besides, a recent study has reported that overexpressing one *AcBCH* of a paralogous gene pair can negatively regulate the expression of the other *AcBCH* of this pair in kiwifruit [23]. Our data showed that in carotenoid-deficient callus Rm, the overexpression of *CsBCH2* upregulated *CsBCH1* to stimulate the increase in carotenoid flux, whereas in transgenic line carotenoid-sufficient callus Rm33 the overexpression of *CsBCH2* downregulated *CsBCH1* to balance the carotenoid biosynthesis of the α-branch and β-branch (Figs 4C and 5E). Overall, the β-cryptoxanthin synthesis capability of CsBCH1 and CsBCH2 was associated with the considerable accumulation of β-cryptoxanthin in citrus fruits, and the distinct gene expression pattern of *CsBCH2* (from *CsBCH1*) could enable it to compensate for the function of *CsBCH1* by providing different enzyme activity. Based on these findings, we speculated that gene compensation might be the evolutionary mechanism for maintaining phenotypic stability when genetic variation or external factors cause the inactivation of *CsBCH1*. Our speculation was supported by the previous report that most duplicated genes are retained during evolution with functional redundancy [39]. The CRISPR/Cas9 system has been successfully used for gene function research in citrus [44]. Considering this, we suggest that the CRISPR/Cas9 system should be employed for future research on loss-of-function mutants of *CsBCH1* or *CsBCH2* to reveal the possible retaining mechanism of the two *CsBCH* genes.

In conclusion, our study indicated that, just like CsBCH1, CsBCH2 could hydroxylate the β-ring of β-carotene, while CsBCH2 exhibited a different and weaker catalytic activity compared with CsBCH1, which could be utilized to provide β-cryptoxanthin substrate in *E. coli* to expand the synthetic biology toolkit for carotenoid engineering. This study revealed the distinct expression pattern and functional characteristics of two *CsBCH* paralogous genes, and illustrated the potential compensatory role of *CsBCH2* for *CsBCH1* in xanthophyll biosynthesis. Our findings provide insights into the mechanism of duplicate β-carotene hydroxylase gene retention during evolution.

## Materials and methods

### Plant materials

The citrus materials Ponkan mandarin (*C. reticulata* Blanco), Hamlin sweet orange (*C. sinensis* Osbeck), and Shatian pummelo (*C. maxima* (Burm.) Merr.) were collected from trees in the orchard of the National Citrus Breeding Center at Huazhong Agricultural University. Citrus calli were derived from Marsh grapefruit (*C. paradisi* Macf. Rm) and ECM Rm33. The calli were subcultured at 25°C on solid Murashige and Tucker (MT) medium. All samples were frozen in liquid nitrogen after collection and stored at −80°C for further analysis.

### RNA extraction and qRT–PCR analysis

Total RNA extraction was performed as previously described [45]. cDNA synthesis was conducted using the HiScript II RT SuperMix for qPCR (+gDNA wiper, Vazyme). The qRT–PCR primers used in this study are listed in Supplementary Data Table S2. The endogenous reference gene was named *CsActin*. qRT–PCR was performed on the Roche LightCycler 480 system (Roche, https://www.roche.com). The qRT–PCR procedure and calculation of the relative expression of genes were carried out as previously described [45, 46]. Each experiment was performed independently with three replicates.

### Gene cloning and sequence analysis

The reference gene sequences of *CsBCH1* (Cs9g19270) and *CsBCH2* (Cs5g03200) were obtained from the Citrus Pan-genome to Breeding Database (http://citrus.hzau.edu.cn/). The full-length coding sequence of *CsBCH1* and *CsBCH2* from the fruits of Ponkan mandarin, Hamlin sweet orange, and Shatian pummelo were amplified using PCR with the primers listed in Supplementary Data Table S1. Multiple sequence alignments were performed using MEGA 6 software, and alignment results were visualized in GeneDoc software.

### Heterologous expression and functional characterization of CsBCH1 and CsBCH2

The ORFs of *CsBCH1* and *CsBCH2* were cloned into vector pHIS8, which was derived from pET28a (+) with modification [47]. To investigate the hydroxylase activity of CsBCH1 and CsBCH2 *in vivo*, pHIS8-CsBCH1, pHIS8-CsBCH2a, pHIS8-CsBCH2a-s, or pHIS8-CsBCH2b-s was co-transformed into *E. coli* BL21 (DE3) with pACCAR16∆crtX. The pHIS8 empty vector was co-transformed with pACCAR16∆crtX into *E. coli* BL21 (DE3) to accumulate β-carotene in host cells as a negative control. Colonies were cultured in Luria–Bertani (LB) medium containing kanamycin (50 mg/ml) and chloramphenicol (25 mg/ml) at 37°C in the dark until the optical density (OD600) reached 0.6. A final concentration of 0.3 mmol/l isopropyl β-d-thiogalactoside (IPTG) was added to induce the expression of CsBCH for 16 hours at 16°C by shaking at 160 rpm. The 50-ml culture was centrifuged at 6000 g for 10 minutes, and the cells were stored at −80°C for further analysis. The experiments were performed with at least three replicates for each sample.

### Carotenoid extraction and analysis

Citrus calli were lyophilized and ground into powder for carotenoid analysis using a Labconco FreeZone lyophilizer. The separation and analysis of carotenoid pigments were performed using HPLC, as previously described [48]. Carotenoids were determined by their specific retention time, absorption spectra, and comparison with authentic standards as previously described [49]. Peak areas were recorded for phytoene, phytofluene, and the other carotenoids at 286, 348, and 450 nm, respectively. The content of carotenoids was quantified using calibration curves for the appropriate standards [48]. The experiment was conducted in triplicate.

### Callus transformation

The coding sequence of *CsBCH2* from the fruits of Ponkan mandarin was cloned into the MT-GFP vector with CaMV35S promoter and enhanced GFP. The stable transformation of citrus callus and growth conditions were as described previously [50]. We performed at least six rounds of callus subculturing on culture media, accompanied by appropriate antibiotics selection. The transgenic calli were collected every 20 days, frozen in liquid nitrogen, and stored at −80°C until further analysis.

### Transmission electron microscopy observation

Calli were subcultured on MT medium for 20 days and collected from transgenic lines. Samples were observed on the electron microscopy platform of Huazhong Agricultural University for subsequent analyses, as previously described [29]. The number and area of PGs per plastid were measured using ImageJ software (version 1.53, NIH, USA), based on the images from TEM. Six plastids were selected randomly from each callus transgenic line.

### Statistical analysis

All data were expressed as the mean ± standard error of three replicates. The statistical analysis of data was performed using GraphPad Prism v.8. Student’s *t*-tests were conducted to determine the statistical significance of differences between transgenic lines and EV.

## Acknowledgements

The authors are very grateful to Prof. Norihiko Misawa for providing the pACCAR16∆ crtX plasmid and grateful to Prof. Robert M. Larkin and Prof. Pengwei Wang for providing expression vectors. We thank Prof. Ping Liu for language improvement. This research was supported by the National Natural Science Foundation of China (No. 31930095 and 32172527) and the Modern Agro-industry Technology Research System (CARS-26).

## Author contributions

X.X.D. supervised the research; Y.Z.Z. and X.X.D. designed the experiments; Y.Z.Z. performed the experiments with contributions from J.J.J.; S.C.Z. and Z.Z.X. provided the plant materials. Y.Z.Z. and X.X.D. wrote the manuscript; Q.S., Y.Z., and J.L.Y. provided critical comments on manuscript editing.

## Data availability

All relevant data are included in the paper and its supplementary files.

## Conflict of interest

The authors declare no competing interests.

## Supplementary data

Supplementary data is available at *Horticulture Research* online.

## Supplementary Material

Web_Material_uhac290
