# Supplementary material for: Citrus β-carotene hydroxylase 2 (BCH2) participates in xanthophyll synthesis by catalyzing the hydroxylation of β-carotene and compensates for BCH1 in citrus carotenoid metabolism
Source: Hortic Res. 2022 Dec 30;10(3):uhac290. doi: 10.1093/hr/uhac290 (PMC10018782; doi:10.1093/hr/uhac290)
Supplement: Web_Material_uhac290 [file web_material_uhac290.zip › Final Revised Supplementary file.docx]

**Supplementary information**

Supplemental Figure 1. FPKM/RPKM of *CsBCH* genes in five citrus tissues from the database.


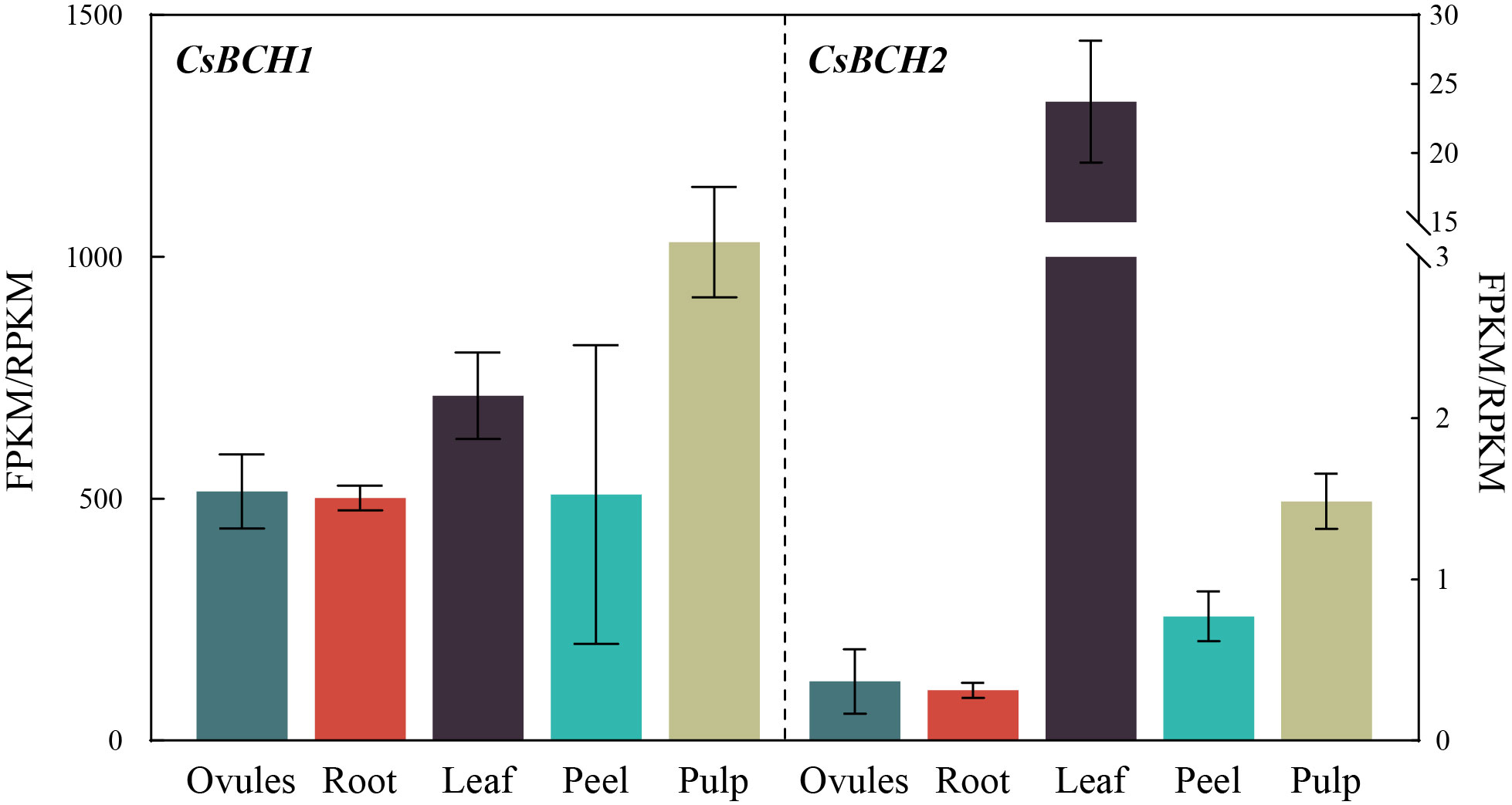
Data published by the Citrus Pan-genome to Breeding Database website. The data are expressed as mean ± standard error (n = 3).

Supplemental Table 1. Primers used for vector construction in this study.

| **Name** | **Primer sequences (5’ →3’)** | **Notes** |
| --- | --- | --- |
| *CsBCH1*-F | TGTGGTCTGCGTCCATTTAA | Gene cloning |
| *CsBCH1*-R | TTTCACGCTCACGCCATTA |  |
| *CsBCH2*-F | ATGGCAAGTGGAATGTCATC |  |
| *CsBCH1-*R | CAGTTGAGCCTTAATTTAGC |  |
| pHIS8-*CsBCH1*-F | CGTGGTTCCCATGGCGGATCCATGCTTTTTGCCCCT | Primers for prokaryotic expression vector construction |
| pHIS8-*CsBCH1*-R | TTGTCGACGGAGCTCGAATTCTTTTGGAACCCTGTTG |  |
| pHIS8-*CsBCH2*-F | CGTGGTTCCCATGGCGGATCCATGGCAAGTGGAATG |  |
| pHIS8-*CsBCH1*-R | TTGTCGACGGAGCTCGAATTCCGTTTCTTTATCTAA |  |
| MT-*CsBCH2*-F | AAGTCCGGAGCTAGCTCTAGAATGGCAAGTGGAATG | Primers for overexpression vector construction |
| MT-*CsBCH2*-R | GCCCTTGCTCACCATGGATCCCGTTTCTTTATCTAA |  |

Supplemental Table 2. Primers used for qRT-PCR in this study.

| **Name** | **Primer sequences (5’ →3’)** |
| --- | --- |
| *CsActin*-qPCR-F | CCAAGCAGCATGAAGATCAA |
| *CsActin*-qPCR-R | ATCTGCTGGAAGGTGCTGAG |
| *CrtB*-qPCR-F | TGGATTCCCAGTGAAGAAGGTC |
| *CrtB*-qPCR-R | CATAATCTGCGCCATCATCAAG |
| *CsBCH2*-qPCR-F | TTTGGGCAAGGTGGGCTCATAG |
| *CsBCH2*-qPCR-R | GAGTCCTGGAACGATGCCTTTG |
| *CsDXS*-qPCR-F | CTCTTCCTTCGCCGTTTCC |
| *CsDXS*-qPCR-R | CAGACCAGCGGCAAAAGTAAC |
| *CsDXR*-qPCR-F | CGATTCTGCTACCCTTTTCAAC |
| *CsDXR*-qPCR-R | ATTGAGTGTATGATAGACTGTGGATG |
| *CsHDS*-qPCR-F | GGTCGGATGAAATCTGCTAT |
| *CsHDS*-qPCR-R | TTCTGGTGGTCCGTAAGTG |
| *CsHDR*-qPCR-F | TCTCTTGAAGGTGTGAGGTATTGC |
| *CsHDR*-qPCR-R | GTTACACAAGAGCGACAAGATGC |
| *CsIPI-*qPCR-F | GGTGAGGAATGCTGCACAAAG |
| *CsIPI*-qPCR-R | AACTCATCAACTGGCACATCTTC |
| *CsGGPPS*-qPCR-F | TAGAGTTCCCTCAGTTACGCACAG |
| *CsGGPPS*-qPCR-R | GCCAGTTCTCTTGTCTTTTGTATCC |
| *CsPSY1*-qPCR-F | CCCGGACTGCTGTGTTTAAT |
| *CsPSY1*-qPCR-R | GAGCAAGGATGCCTCAAATC |
| *CsPDS*-qPCR-F | ATAATTGGCGGACAGGCATA |
| *CsPDS*-qPCR-R | CCTCTGTCGTCACTCGATCA |
| *CsZDS*-qPCR-F | ATCAGTGCTCGTTGTATGCTTACTATATT |
| *CsZDS*-qPCR-R | CCCTTGAGCATCCGCAAT |
| *CsLCYE*-qPCR-F | CAACTGGATATTGAGGGCATCA |
| *CsLCYE*-qPCR-R | CAAGGAAACCGTGCCACATC |
| *CsLCYB1*-qPCR-F | GGCTATATGGTGGCAAGGACTT |
| *CsLCYB1*-qPCR-R | CAGAATTGAGGCTTCGAACGA |
| *CsLCYB2*-qPCR-F | CCCTATTTCCATTAGGCCGC |
| *CsLCYB2*-qPCR-R | CACGTCATATCGAATACGATC |
| *CsBCH1*-qPCR-F | TTTGGGATGGCCTACATGTTC |
| *CsBCH1*-qPCR-R | GGCACGTCGGCAATGG |
| *CsCYP97C1*-qPCR-F | TCTATGTTAGTTGCGGGCCA |
| *CsCYP97C1*-qPCR-R | GGAGGATGTGGGTAGAGACG |
| *CsCYP97A3*-qPCR-F | AGAGGCGAAAGGGACAATCA |
| *CsCYP97A3*-qPCR-R | ACGCCATATCTCCCCATCTG |
| *CsZEP*-qPCR-F | GAAGCAATTCTTCGACGTGACA |
| *CsZEP*-qPCR-R | ACCGAGTCCCCAAGCAAAGT |

Supplemental Table 3. Composition and content of carotenoids in *CsBCH2-*overexpressing transgenic line callus Rm*.*

| **Carotenoids** | **Sample** | **Content (ug/g DW)**^a^ |
| --- | --- | --- |
| Antheraxanthin-isomer | Rm: EV  OE-1  OE-3  OE-6 | ND  1.04 ± 0.60^***^  1.72 ± 0.99^***^  1.54 ± 0.89^***^ |
| Lutein | Rm: EV  OE-1  OE-3  OE-6 | 5.61 ± 0.69  0.78 ± 0.45^***^  4.38 ± 2.53  3.85 ± 2.28 |
| Violaxanthin | Rm: EV  OE-1  OE-3  OE-6 | 1.20 ± 0.03  9.29 ± 1.18^**^  10.77 ± 1.13^**^  9.19 ± 5.31^**^ |
| Violaxanthin-isomer1 | Rm: EV  OE-1  OE-3  OE-6 | ND  1.05 ± 0.61^***^  1.73 ± 0.99^***^  1.55 ± 0.89^***^ |
| Violaxanthin-isomer2 | Rm: EV  OE-1  OE-3  OE-6 | ND  1.04 ± 0.60^***^  2.23 ± 1.29^***^  1.94 ± 1.12^***^ |
| Total | Rm: EV  OE-1  OE-3  OE-6 | 6.81 ± 0.72  6.20 ± 0.44  21.67 ± 0.97^**^  18.17 ± 3.46^**^ |

^a^The data were expressed as mean ± standard error (n = 3); ND, not detected; DW, dry weight. Asterisks indicate statistically significant differences compared with EV (Student’s t-test P-value; **, P < 0.01, ***, P < 0.001).

Supplementary Table 4. Composition and content of carotenoids in *CsBCH2-*overexpressing transgenic line callus Rm33*.*

| **Carotenoids** | **Sample** | **Content (ug/g DW)**^a^ |
| --- | --- | --- |
| *α*-carotene | Rm33:EV  OE-3  OE-6  OE-7 | 47.42 ± 0.91  ND  ND  ND |
| *β*-Carotene | Rm33:EV  OE-3  OE-6  OE-7 | 83.58 ± 2.14  ND  ND  ND |
| Antheraxanthin-isomer | Rm33:EV  OE-3  OE-6  OE-7 | ND  1.71 ± 0.08^**^  1.45 ± 0.07^**^  1.72 ± 0.10^**^ |
| Lutein | Rm33:EV  OE-3  OE-6  OE-7 | 52.17 ± 1.58  23.13 ± 0.82^***^  16.78 ± 1.67^**^  17.15 ± 1.71^***^ |
| Phytoene | Rm33:EV  OE-3  OE-6  OE-7 | 10.75 ± 1.20  26.13 ± 0.44^**^  10.58 ± 0.52  15.06 ± 1.28 |
| Phytofluene | Rm33:EV  OE-3  OE-6  OE-7 | 1.48 ± 0.02  4.15 ± 0.03^**^  1.89 ± 0.15  2.61 ± 0.26 |
| Phytofluene-isomer | Rm33:EV  OE-3  OE-6  OE-7 | 0.70 ± 0.07  1.38 ± 0.04^**^  0.69 ± 0.01  0.92 ± 0.08 |
| Violaxanthin | Rm33:EV  OE-3  OE-6  OE-7 | ND  14.85 ± 0.28^***^  9.29 ± 1.18^**^  10.77 ± 1.13^**^ |
| Violaxanthin-isomer1 | Rm33:EV  OE-3  OE-6  OE-7 | ND  1.38 ± 0.03^***^  1.02 ± 0.02^***^  1.08 ± 0.06^***^ |
| Violaxanthin-isomer2 | Rm33:EV  OE-3  OE-6  OE-7 | ND  2.20 ± 0.03^***^  1.68 ± 0.10^***^  1.84 ± 0.12^***^ |
| Total | Rm33:EV  OE-3  OE-6  OE-7 | 196.09 ± 4.92  75.10 ± 0.58^**^  43.71 ± 2.21^***^  48.90 ± 3.50^***^ |

^a^The data were expressed as mean ± standard error (n = 3); ND, not detected; DW, dry weight. Asterisks indicate statistically significant differences compared with EV (Student’s t-test P-value; **, P < 0.01, ***, P < 0.001).
